# Prevalence and associated factors of HIV among female sex workers in Eastern and Southern Africa: Systematic review and meta-analysis

**DOI:** 10.1371/journal.pone.0313868

**Published:** 2024-12-02

**Authors:** Tigabu Kidie Tesfie, Getaneh Awoke Yismaw, Bantie Getnet Yirsaw, Habtamu Wagnew Abuhay, Meron Asmamaw Alemayehu, Nebiyu Mekonnen Derseh, Gebrie Getu Alemu, Muluken Chanie Agimas

**Affiliations:** Department of Epidemiology and Biostatistics, Institute of Public Health, College of Medicine and Health Sciences, University of Gondar, Gondar, Ethiopia; Universidad Nacional Autónoma de México Facultad de Medicina: Universidad Nacional Autonoma de Mexico Facultad de Medicina, MEXICO

## Abstract

**Background:**

Female sex workers (FSWs) are marginalized groups of the population who have limited access to healthcare and a higher risk of HIV infection due to biobehavioral and structural risk factors. Although it is known that the Eastern and Southern African region is affected by the highest HIV incidence and prevalence, understanding the burden among FSWs in the region remains limited. We aimed to assess the pooled prevalence and associated factors of HIV in this vulnerable population.

**Methods:**

Relevant studies were searched on PubMed, Embase, Scopus, ScienceDirect, Hinari, African Journals Online (AJOL), Google, and Google Scholar. The searching mechanism was constructed using keywords identified by CoCoPop (Condition, Context, and Population) framework and medical subject heading terms to recruit studies published between January 1, 2015 and March 30, 2024. Observational studies that estimate the prevalence or incidence or associated factors of HIV among FSWs, even if FSWs were not the main focus of the study, were included. The quality of included studies was assessed using Joana Brigg’s Institute checklist. Data were extracted and analyzed using STATA 17 software. To estimate the pooled effect sizes with their 95% confidence intervals, a random effect model was fitted. The I^2^ statistic was used to evaluate heterogeneity. Funnel plot and Egger’s regression test were utilized to assess the small study effect. Publication bias was managed using trim-and-fill analysis. Subgroup and sensitivity analysis were considered to handle heterogeneity among studies.

**Results:**

A total of 54 articles with 78,747 FSWs who had successful HIV biological test were included in the analysis. The pooled prevalence of HIV among FSWs in Eastern and Southern Africa was 36.0% (95% CI: 31.0%–41.0%). Regionally, the pooled prevalence was 59.0% (95% CI: 53.0%–64.0%) and 29.0% (95% CI: 25.0%–34.0%) in Southern and Eastern Africa, respectively. Age above 35 (POR = 7.35; 95% CI: 5.26, 10.28) and 25–34 years (POR = 2.91; 95% CI: 2.02, 4.21), being married (POR = 1.33; 95% CI: 1.07, 1.66), divorced (POR = 1.72; 95% CI: 1.39, 2.13), and widowed (POR = 2.70; 95% CI: 2.09, 3.49), primary education (POR = 1.29; 95% CI:1.08, 1.55), history of sexually transmitted infection (POR = 1.51; 95% CI: 1.20, 1.90), syphilis (POR = 2.57; 95% CI: 1.66, 3.98), Hepatitis B infection (POR = 2.60; 95% CI: 1.07,6.32), drinking alcohol (POR = 1.21; 95% CI: 1.01, 1.45) and injectable drug use before sex (POR = 1.75; 95% CI: 1.24, 2.47) were found significantly associated with the increased risk of HIV infection among FSWs.

**Conclusion:**

These data suggest an urgent need to improve access to HIV prevention programs for FSWs. Providing behavioral interventions including reduction of alcohol and injectable drug use before sex, initiating tailored healthcare services, strengthening the psychosocial and legal support network, and fostering partnership might reduce the burden in the region. Clinicians could facilitate early detection and treatment of STIs, and Hepatitis B virus infection.

**Trial registration:**

**Protocol registration:** The protocol for this systematic review and meta-analysis was registered in the PROSPERO with registration number CRD42024509200.

## Introduction

HIV continues to be one of the most pressing global health issues, impacting millions of people globally. The Joint United Nations Programme on HIV/AIDS (UNAIDS) report released on World AIDS Day 2023 states that, since the epidemic’s beginning, 85.6 million people have contracted HIV globally, and 40.4 million of those individuals have passed away from AIDS-related illnesses. In 2022, 39 million people were living with HIV, and 1.3 million people were newly infected with the virus globally. In the same year, women and girls accounted for 46% of incident HIV infections and 53% of all HIV-positive individuals. Globally, in 2022, 86% and 76% of individuals living with HIV were aware of their status and were receiving antiretroviral medication, respectively. Sub-Saharan Africa is the most HIV-affected region in the world, home to over 70% of all HIV-positive individuals worldwide. The Eastern and Southern African countries were affected by the highest incidence and prevalence of HIV throughout the world, in which 20.8 million people were living with HIV and 260,000 new infections occurred in 2022 [1].

HIV has many adverse effects on individual, family, and national socio-economic status because of the loss of productive human power, decreased participation in formal education, and over-expenditure for healthcare-seeking [2,3]. The primary motivation for the worldwide response to the HIV epidemic, which involves resource mobilization, campaigning, and international collaboration, is to address the disease’s multifaceted effects across several decades with the main goal of putting an end to the epidemic. These global response strategies have resulted in significant progress in the fight against HIV [4].

Despite the scale‐up in the effective and evidence‐based interventions for HIV testing, prevention, and treatment, which was witnessed by global declines in HIV incidence from 3.1 million in 2002 to 2.7 million in 2010, and 1.3 million in 2022 [1,5], several challenges are persisting towards HIV prevention, testing, and treatment services. Stigma and discrimination, limited access to health care services, and difficulty in reaching key populations are the main challenges that hamper HIV prevention, testing, treatment initiation, and adherence [6]. The world is aspiring to eradicate AIDS by 2030; however, it will be challenging due to the significant prevalence and incidence of the infection [7].

According to existing evidence, HIV incidence is highly contributed by key populations [8]. Key populations, including female sex workers (FSWs), men who have sex with men, people who inject drugs, people in prisons, and transgender individuals are groups of individuals who have higher risk of HIV due to their specific risky behaviors [9]. These individuals are marginalized groups who lack access to HIV counselling, testing, and treatment services [10,11]. Existing disparities in access to HIV treatment and prevention services for those key populations impede the global response to HIV. Even though they are a small proportion of the general population, in 2019, more than 60% of new adult HIV infections globally were recorded among key populations and their sexual partners [12]. In Western and Central Africa, 69% of new infections are concentrated in key populations, and sex workers have a 30-fold increased risk of HIV infection [12]. Among key populations, FSWs have the double burden of being women and belonging to the key population group for risk of HIV infection [11].

Worldwide, FSWs carry a disproportionately large burden of HIV infection [13]. They are a key population for engaging in HIV programs to both improve these women’s health by reducing their risk of acquiring HIV infection through preventive measures and halting the ongoing HIV transmission from the stem. The worldwide prevalence of HIV among FSWs was 11.8%. FSWs have more than 13.5 times increased odds of HIV infection as compared to the general reproductive-age women in low- and middle-income countries. When compared to other regions, sub-Saharan Africa holds the highest HIV prevalence among FSWs, with nearly 40% of FSWs living with HIV [14,15]. Estimation of HIV infection in FSWs is critical to monitor the progress in the response among communities most often overlooked by treatment and prevention programs [16]. Even though HIV prevention and treatment programs were expanded, the highest HIV burden in Eastern and Southern Africa is needing concerted efforts [17]. Efforts should be redoubled in the region to achieve the UNAIDS fast-track targets by 2030. The priority must focus on detection, treatment, and virological suppression of all existing HIV infections in the region [18]. Hence, knowing the extent of the epidemic among FSWs is crucial to designing targeted interventions. In the setting, these vulnerable groups of populations contribute significantly to the spread of HIV infection due to their frequent exposure to violence, multiple clients, higher mobility, stigma, criminalization, and harmful substance use as a result of poverty and low education level [27]. To control the highest incidence of HIV in the context, preventive interventions involving FSWs are urgently needed.

Because of behavioral and structural risk factors, there is a substantial risk of HIV transmission among FSWs. The behavioral risk factors like having multiple sexual partners, inconsistent condom use, sexual violence from partners, and involvement in high-risk sexual practices increase the risk of HIV transmission. In addition, there is a greater risk of sexually transmitted infections (STIs) among FSWs, which may have contributed to the elevated risk of HIV infection. Injectable drug use and alcohol consumption were high among FSWs as compared to other women, which plays a role in practicing sex without condoms and sharing needles and injection equipment with others [11,19–21]. The structural risk factors include the working environment, stigma and discrimination, poverty, and criminalization of sex work, which can increase the risk for HIV infection among FSWs through creating barriers to HIV care and prevention service accessibility [20,22,23]. The settings of sex work have a large impact on increasing vulnerability for infection through hardening the ways to negotiate use of condoms, violence protection, and access to HIV treatment and prevention services [24]. In addition, laws that aim to criminalize FSWs have created obstacles to accessing preventive services [25].

Addressing HIV among FSWs needs sustained empowerment and engagement of the community, continued investigations and research, political support, innovative policies, and structural programs [26]. Despite the availability of well-established evidence indicating that Southern and Eastern Africa has the highest incidence and prevalence of HIV in the world [1], to our knowledge, there is no comprehensive evidence on the pooled prevalence and associated factors of HIV among FSWs. The need for local research to ensure targeted and efficient HIV response in Eastern and Southern Africa, as indicated by previously published evidence [18], is the main driver of the study. Even though FSWs are key sources for incident HIV infection in the setting, there is little emphasis on targeting those populations for HIV prevention and treatment services [27]. Understanding the overall prevalence of HIV and its associated factors will help in shaping policy directions, setting priorities, and providing targeted interventions in the region. Therefore, the aim of this systematic review and meta-analysis is to determine the pooled prevalence and associated factors of HIV infection among FSWs in Eastern and Southern Africa.

## Methods

### Protocol registration and reporting

At the inception stage of this systematic review and meta-analysis, a preliminary search and idea validation were done before protocol development in order to ensure the rationality of the proposed idea, avoid duplication of previously addressed problems, and assure the availability of enough articles for the review. The protocol for this systematic review and meta-analysis was registered in the international database of the Prospective Register of Systematic Reviews (PROSPERO) with registration number CRD42024509200. The search strategy, selection of studies, extraction of data, and result reporting were done following the Preferred Reporting Items for Systematic Reviews and Meta-Analyses (PRISMA) 2020 statement [28].

### Searching strategy

A comprehensive literature search was done on PubMed, Embase, Scopus, ScienceDirect, Hinari, African Journals Online (AJOL), Google, and Google Scholar to retrieve relevant articles. The reference list of pertinent articles was also searched to identify additional studies. Two independent reviewers (TKT and MCA) searched the databases from February 8 to March 30, 2024. For instance, the following combination was used for searching in PubMed: (Prevalence OR Magnitude OR Proportion OR burden OR Epidemiology) AND (HIV OR HIV/AIDS OR AIDS Virus OR Acquired Immune Deficiency Syndrome Virus)) OR (HIV OR HIV/AIDS OR AIDS Virus OR Acquired Immune Deficiency Syndrome Virus[MeSH Terms])) AND (Female Sex worker OR commercial sex worker OR sex worker OR prostitute OR brothel worker OR transaction* sex worker)) AND (Factors OR “Associated factors” OR Determinants OR Predictors OR “Related factors” OR “Risk factor*”)) AND (Eritrea OR Ethiopia OR Djibouti OR Somalia OR South Sudan OR Kenya OR Uganda OR Rwanda OR Burundi OR Tanzania OR Malawi OR Zambia OR Zimbabwe OR Mozambique OR Seychelles OR Comoros OR Madagascar OR Mauritius OR Eswatini OR Swaziland OR Lesotho OR Botswana OR South Africa OR Namibia). Manual searching (general search) was done using Google and Google Scholar search engines. The search strategy includes keywords identified from the CoCoPop (Condition, Context, and Population) framework, MeSH headings, and entry terms identified through the MeSH browser and brainstorming. The synonyms and keywords were connected using “OR” and “AND” Boolean operators. Searching strategies utilised in different databases are shown in S1 File.

### Eligibility criteria

Observational studies (cross-sectional or cohort) conducted in any of Eastern and Southern African countries that were published in English language, reporting the prevalence and/or associated factors of HIV among FSWs even if they were conducted with another primary purpose, and published between January 1, 2015, and March 30, 2024, were included. However, experimental studies, qualitative studies, and observational studies that report HIV status of participants based on the self-report of FSWs, studies with incomplete information on both prevalence and associated factors, duplicate reports of HIV prevalence, and studies without full texts were excluded. The review question was formulated based on the CoCoPop framework for prevalence studies as follows:

**Condition**: HIV (prevalence and associated factors)
**Context**: Eastern and Southern African countries and
**Population**: FSWs

### Outcome of interest

Our main (primary) outcome of the review was the prevalence of HIV among FSWs in Eastern and Southern African countries. In this analysis, the prevalence or cumulative incidence of HIV that was estimated based on the biological assessment of blood samples for the presence or absence of the virus was used. In addition, we also considered factors associated with HIV among FSWs as an additional (secondary) outcome of the review.

### Study screening and selection

After developing a comprehensive search strategy with the agreement of two authors (TKT and MCA), titles and abstracts of studies were retrieved using the searching strategies. Then titles and abstracts of studies were exported to EndNote-citation manager software and screened independently after duplicates were removed. The full texts of those potentially eligible studies were downloaded and screened by the two review authors for final inclusion. Any disagreement by review authors regarding the eligibility of studies was resolved through discussions involving a third author (NMD).

### Study quality assessment

Two review authors (TKT & GAY) independently assessed the quality of studies by considering different characteristics of the study to minimise errors in assessments and to provide judgements not influenced by a single person’s preconceptions. To improve the reliability of assessments, we discussed the use of the assessment tool, and we used a piloted risk-of-bias assessment tool on a sample of articles, which helped us to be consistent and to reach consensus easily. Then the above two review authors discussed the discrepancies in quality assessment and reached an agreement. If this fails to reach an agreement, the third author (BGY) was involved, and agreements were reached with reasonable justification. The Joanna Brigg’s Institute (JBI) quality assessment checklist for cross-sectional and cohort studies was utilized. The JBI quality assessment checklist had eight and eleven items for cross-sectional and cohort studies, respectively [29]. The checklists were used to assess the risk of bias related to recruitment of participants, measurement of outcome and independent variables, and analysis. Each item has either “yes,” “no,” or “unclear” answers. Scoring was made based on the total number of “yes” answers. For cross-sectional studies, articles scoring 7–8, 4–6, and 0–3 “yes” points were classified as having low, moderate, and high risk of bias, respectively. For cohort studies, articles scoring 9–11, 5–8, and 0–4 “yes” points were categorised as having low, moderate, and high risk of bias, respectively [30]. See S1 File for results of the study quality assessment based on the JBI’s critical appraisal checklists.

### Data extraction

We used a pre-piloted data extraction Excel sheet to extract the data from the final included studies for narrative synthesis and meta-analysis. We extracted the data based on the study characteristics (last name of first author, year of publication, country, region, study design, sample size and events or number of HIV-positive FSWs), participant’s characteristics (age, sexually transmitted infections, alcohol and drug use status, condom use and breakage), and quality assessment. Two independent review authors (TKT & MCA) extracted the data with the involvement of a third author (GAY) during discrepancies.

### Data processing and analysis

The extracted data was exported to STATA/MP version 17 for further statistical analysis. A narrative summary for all included studies was provided. During the narrative summary (systematic review), all of the eligible articles were summarised according to publication year, country, region, study design, and sample size. The number of FSWs who had HIV (events) and total number of study participants with successful HIV testing were considered to calculate the pooled prevalence (proportion) of HIV in the meta-analysis. Then, using the “***metaprop***” STATA command, the pooled prevalence was estimated. The pooled prevalence of HIV among FSWs and other factors were reported by text description and forest plot. To estimate the pooled odds ratio, initially, the log of the odds ratio and the standard error of the log odds ratio were calculated. Then, using the “***metan***” command, the pooled log odds ratio was estimated and transformed into a pooled odds ratio (POR). First, we fitted a fixed effect meta-analysis model, then we assessed heterogeneity between included studies by I^2^ statistics. Statistically significant heterogeneity between studies was determined using Higgins’ I^2^ statistics when p-value <0.05 at 95% confidence interval, and it was regarded as high, moderate, or low when I^2^ test statistics results were 75%, 50%, and 25%, respectively [31]. Since heterogeneity was high (I^2^ > 75%), random effect (DerSimonian-Laird) meta-analysis was used to determine the pooled estimates in order to account for this substantial heterogeneity. Subgroup analysis was done by study period, region, risk of bias, and study design. Publication bias was assessed and presented using a funnel plot graphically and using Egger’s statistical test. A p-value of less than 0.05 during egger’s regression was considered to declare the presence of publication bias [32]. Trim-and-fill analysis was conducted for the small study effect. In addition, a leave-one-out sensitivity analysis was carried out to detect the source of heterogeneity using the “***metaninf***” command. Results were presented in the form of texts, tables, and figures.

### Ethical consideration

Published articles were used for further analysis, which is systematic review and meta-analysis. Hence, participants were articles. Due to this, consent was not obtained from the study participants.

## Results

### Searching results and study selection

**Fig 1:**
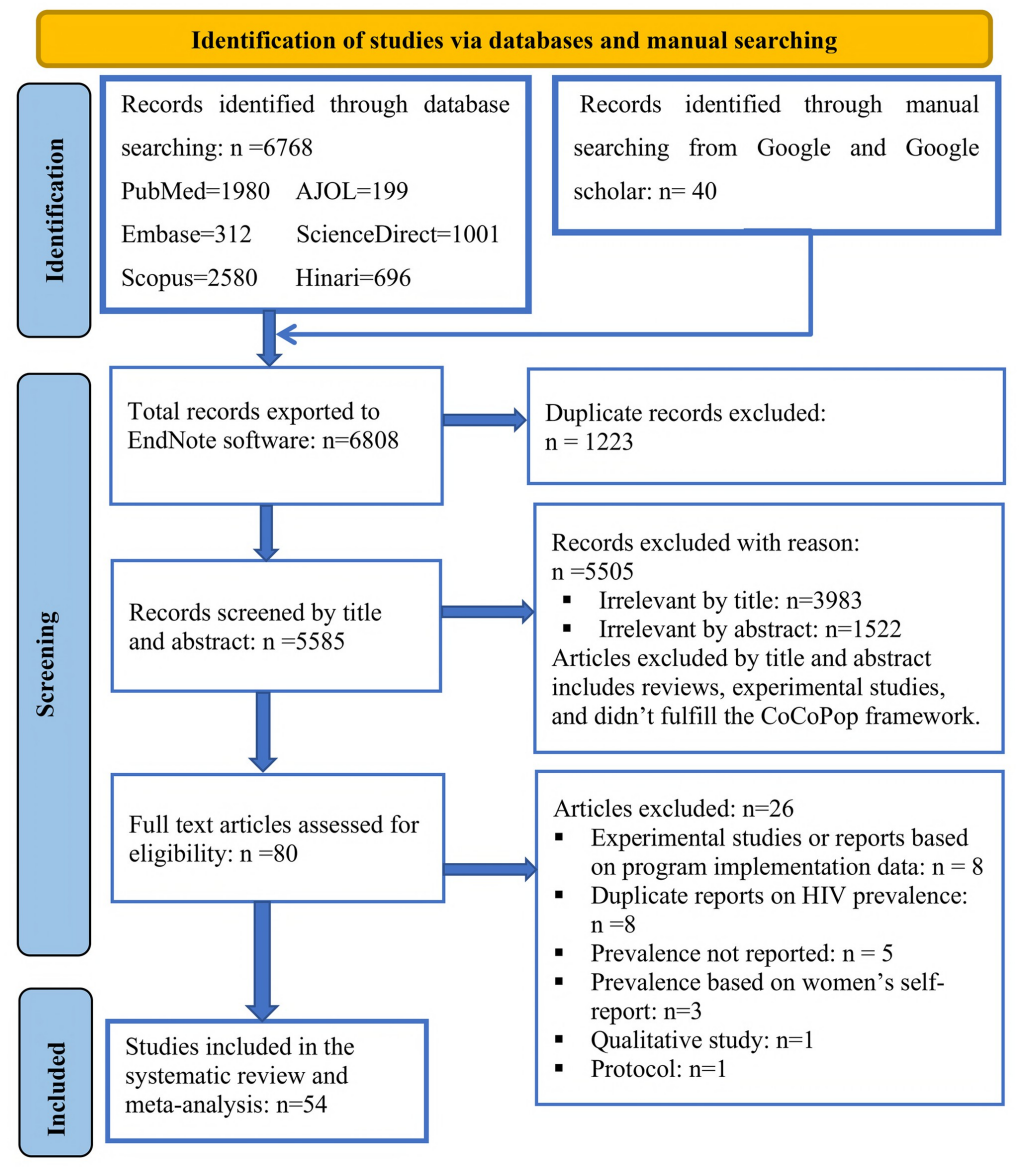
PRISMA flowchart describing identification, screening, and inclusion of articles for systematic review and meta-analysis on pooled prevalence and associated factors of HIV among female sex workers in Eastern and Southern Africa.

The combined literature search strategy retrieved a total of 6808 records. Literature searching was performed through PubMed, Embase, Scopus, ScienceDirect, Hinari, AJOL, Google, and Google Scholar. After the removal of 1223 duplicate records, 5585 records were screened by their title and abstract, of which 5505 articles were irrelevant for the review in terms of the outcome of interest. Then, full texts of 80 studies were assessed, and 54 records were considered suitable for inclusion in this systematic review and meta-analysis based on pre-defined eligibility criteria. Out of the 26 articles excluded by screening full texts, eight were experimental studies, eight studies were duplicate reports on HIV prevalence, in five studies prevalence wasn’t reported, three prevalence studies were based on women’s self-report, one article was a protocol, and one article was a qualitative study (Fig 1).

### Quality assessment

Overall, the included studies were of reasonable quality. More than nine-tenths (90.7%) of included studies had low risk of bias, and the rest five articles (9.3%) had moderate risk of bias. There wasn’t an article judged as having a high risk of bias. Therefore, all selected studies were included in the analysis (S1 File).

### Characteristics of included studies

A total of 54 studies conducted in 14 countries with a total of 78,747 participants who had successful HIV biological tests were included in the analysis. The minimum and maximum sample sizes of included studies were 91 [33] and 9906 [34], respectively. Among the 54 studies included in the analysis, five studies (9.3%) were cohort [35–39] in terms of study design, whereas the rest 49 (90.7%) studies were cross-sectional. Regarding country, nine (16.7%) and eight (14.8%) studies were conducted in Ethiopia [27,33,40–46] and South Africa [35,47–53], respectively. One study was conducted in the cross-border areas of four countries in East Africa (Uganda, Kenya, Rwanda, and Tanzania) [54]. In terms of region, 42 (77.8%) studies were conducted in Eastern Africa. Twenty-nine (53.7%) studies were published between 2015 and 2019, and 25 (46.3%) studies were published from 2020 to 2024 (Table 1).

**Table 1:** Characteristics of included studies for a systematic review and meta-analysis on pooled prevalence and associated factors of HIV among female sex workers in Eastern and Southern Africa (n = 54).

Authors	Publication year	Country	Region	Study design	Sample size	Events	Risk of bias
**Abdella *et al*** [40]	2022	Ethiopia	East Africa	cross-sectional	6085	1138	Low
**Afzal *et al*** [47]	2017	South Africa	Southern Africa	cross-sectional	97	32	Moderate
**Alemu *et al*** [27]	2022	Ethiopia	East Africa	cross-sectional	381	76	Low
**Ali et al** [34]	2022	Zimbabwe	East Africa	cross-sectional	9906	4724	Low
**Amogne *et al*** [41]	2021	Ethiopia	East Africa	cross-sectional	4900	1172	Low
**Ashu *et al*** [42]	2021	Ethiopia	East Africa	cross-sectional	276	34	Low
**Augusto *et al*** [55]	2016	Mozambique	East Africa	cross-sectional	1240	341	Low
**Beattie *et al*** [56]	2024	Kenya	East Africa	cross-sectional	1003	257	Low
**Bolo *et al*** [57]	2023	South Sudan	East Africa	cross-sectional	1,284	146	Low
**Bossard *et al*** [58]	2022	Malawi	East Africa	cross-sectional	363	191	Low
**Cafo *et al*** [33]	2023	Ethiopia	East Africa	cross-sectional	91	8	Moderate
**Chabata *et al*** [59]	2019	Zimbabwe	East Africa	cross-sectional	656	236	Low
**Chanzu *et al*** [60]	2015	Kenya	East Africa	cross-sectional	280	92	Low
**Choudhry *et al*** [61]	2015	Uganda	East Africa	cross-sectional	104	12	Low
**Coetzee *et al*** [48]	2017	South Africa	Southern Africa	cross-sectional	508	272	Low
**Doshi *et al*** [62]	2018	Uganda	East Africa	cross-sectional	1,492	485	Low
**Eakle *et al*** [35]	2017	South Africa	Southern Africa	cohort	692	339	Moderate
**Faini *et al*** [36]	2022	Tanzania	East Africa	cohort	773	59	Low
**Gelan *et al*** [43]	2023	Ethiopia	East Africa	cross-sectional	297	50	Low
**Bugssa *et al*** [44]	2015	Ethiopia	East Africa	cross-sectional	319	38	Low
**Goldenberg *et al*** [63]	2016	Uganda	East Africa	cross-sectional	400	135	Low
**Grosso *et al*** [64]	2018	Lesotho	Southern Africa	cross-sectional	743	534	Low
**Hakim *et al*** [65]	2022	South Sudan	East Africa	cross-sectional	838	414	Low
**Hakim *et al*** [66]	2020	South Sudan	East Africa	cross-sectional	838	317	Low
**Hensen *et al*** [67]	2019	Zimbabwe	East Africa	cross-sectional	2387	563	Low
**Herce *et al*** [68]	2018	Malawi	East Africa	cross-sectional	106	66	Low
**Hladik *et al*** [69]	2017	Uganda	East Africa	cross-sectional	942	323	Low
**Ingabire *et al*** [70]	2019	Rwanda	East Africa	cross-sectional	1168	587	Moderate
**Inguane *et al*** [71]	2015	Mozambique	East Africa	cross-sectional	1240	341	Low
**Jonas *et al*** [72]	2020	Namibia	Southern Africa	cross-sectional	1188	487	Moderate
**Jones *et al*** [37]	2023	Zimbabwe	East Africa	cohort	6665	441	Low
**Kassanjee *et al*** [49]	2022	South Africa	Southern Africa	cross-sectional	2999	1862	Low
**Kerrigan *et al*** [73]	2017	Tanzania	East Africa	cross-sectional	496	203	Low
**Kilembe *et al*** [74]	2019	Zambia	East Africa	cross-sectional	1377	576	Low
**Lancaster *et al*** [75]	2016	Malawi	East Africa	cross-sectional	200	138	Low
**McKinnon *et al*** [38]	2015	Kenya	East Africa	cohort	5668	1717	Low
**Merrigan *et al*** [76]	2015	Botswana	Southern Africa	cross-sectional	947	586	Low
**Metaferia *et al*** [45]	2021	Ethiopia	East Africa	cross-sectional	360	27	Low
**Milovanovic *et al*** [50]	2023	South Africa	Southern Africa	cross-sectional	2999	1862	Low
**Mizinduko *et al*** [77]	2020	Tanzania	East Africa	cross-sectional	952	146	Low
**Moazzami *et al*** [78]	2020	Lesotho	Southern Africa	cross-sectional	743	534	Low
**Mulholland *et al*** [54]	2022	Multi-country	East Africa	cross-sectional	715	85	Low
**Musyoki *et al*** [79]	2015	Kenya	East Africa	cross-sectional	596	183	Low
**Mutagoma *et al*** [80]	2017	Rwanda	East Africa	cross-sectional	1909	819	Low
**Mutagoma *et al*** [81]	2017	Rwanda	East Africa	cross-sectional	1112	567	Low
**Nzivo *et al*** [82]	2019	Kenya	East Africa	cross-sectional	268	44	Low
**Okiria *et al*** [83]	2023	South Sudan	East Africa	cross-sectional	408	108	Low
**Rhead *et al*** [84]	2018	Zimbabwe	East Africa	cross-sectional	173	91	Low
**Rossouw *et al*** [51]	2023	South Africa	Southern Africa	cross-sectional	401	257	Low
**Sweet *et al*** [39]	2020	Kenya	East Africa	cohort	348	84	Low
**Rwema *et al*** [52]	2019	South Africa	Southern Africa	cross-sectional	410	261	Low
**Vu *et al*** [85]	2018	Tanzania	East Africa	cross-sectional	1909	535	Low
**Wariso *et al*** [46]	2023	Ethiopia	East Africa	cross-sectional	6,085	1107	Low
**Wells *et al*** [53]	2018	South Africa	Southern Africa	cross-sectional	410	261	Low

### Pooled prevalence of HIV among female sex workers

**Fig 2:**
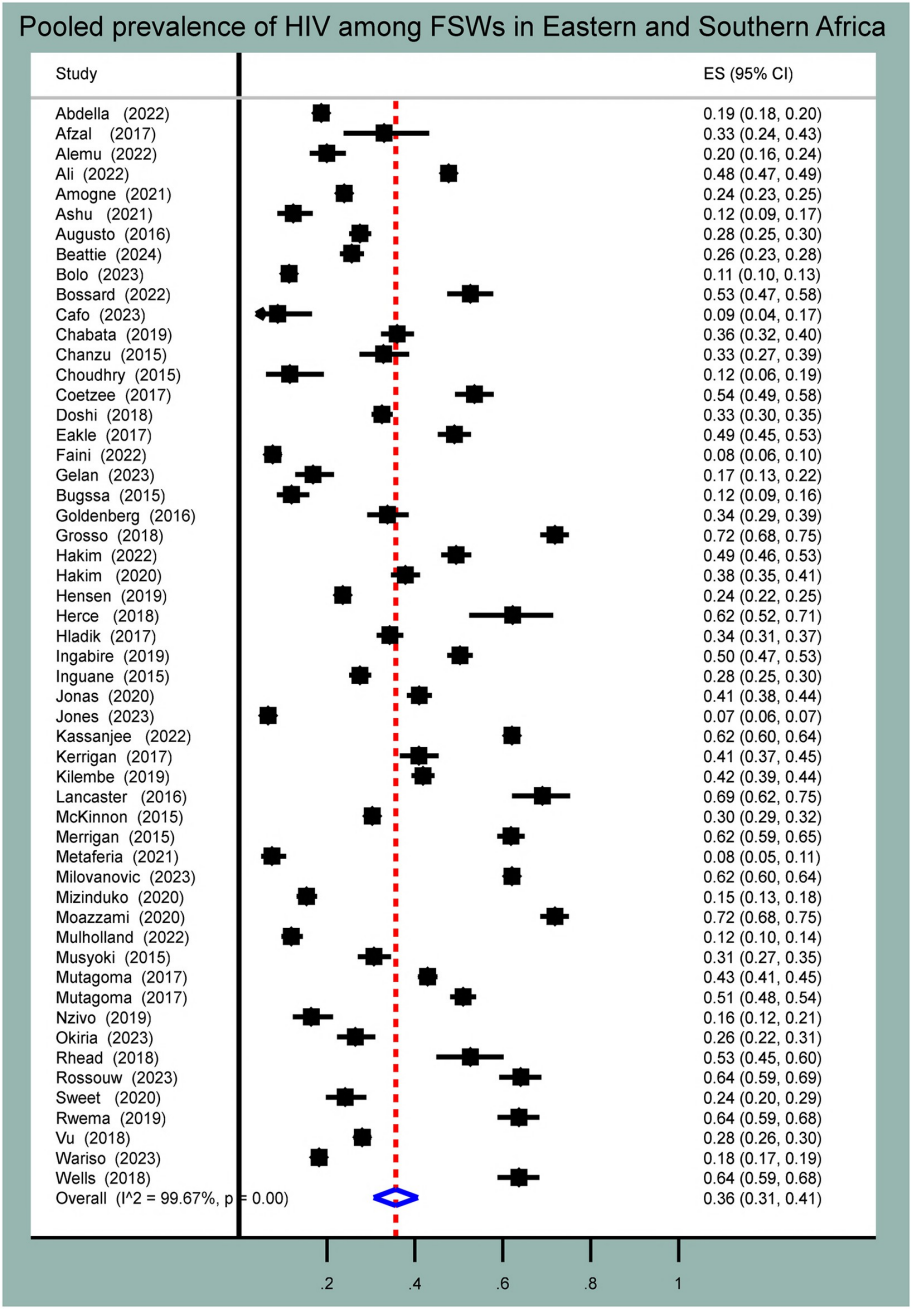
Forest plot for pooled prevalence of HIV among female sex workers in Eastern and Southern Africa.

As the random effect model showed, the pooled prevalence of HIV among female sex workers in Eastern and Southern African countries was 36.0% (95% CI: 31.0%–41.0%). There was high and statistically significant heterogeneity among studies (I^2^ = 99.7%, p-value < 0.001) (Fig 2). Hence, to account for substantial heterogeneity, a random effect model (DerSimonian-Laird method) was used to determine the pooled estimates. Based on Egger’s test and funnel plot, there was publication bias in the included studies (bias coefficient = 13.0, 95% CI: 5.6–20.4, p-value = 0.001). Since there was a small study effect, trim-and-fill analysis was conducted as shown in S1 File.

### Handling heterogeneity

#### Subgroup analysis

**Fig 3:**
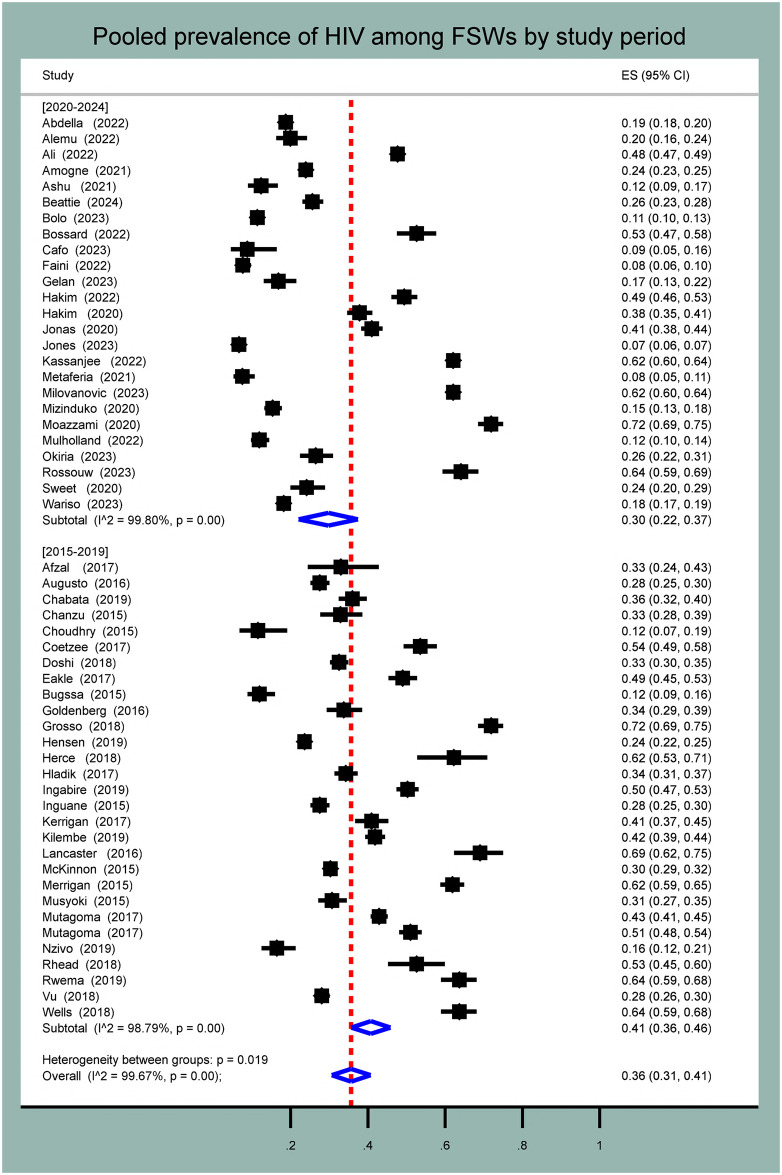
Forest plot for pooled prevalence of HIV among female sex workers in Eastern and Southern Africa by publication year.

We performed sub-group analysis using predetermined parameters to compare the pooled prevalence across subgroups. It was conducted based on publication year, region, study design, and risk of bias. Using publication year, the pooled prevalence of HIV from 2015 to 2019 was 41% (95% CI: 36.0%–46.0%, I^2^ = 98.8%, p-value < 0.001), whereas it was 30% (95% CI: 22.0%–37.0%, I^2^ = 99.8%, p-value < 0.001) from 2020–2024 (Fig 3).

**Fig 4:**
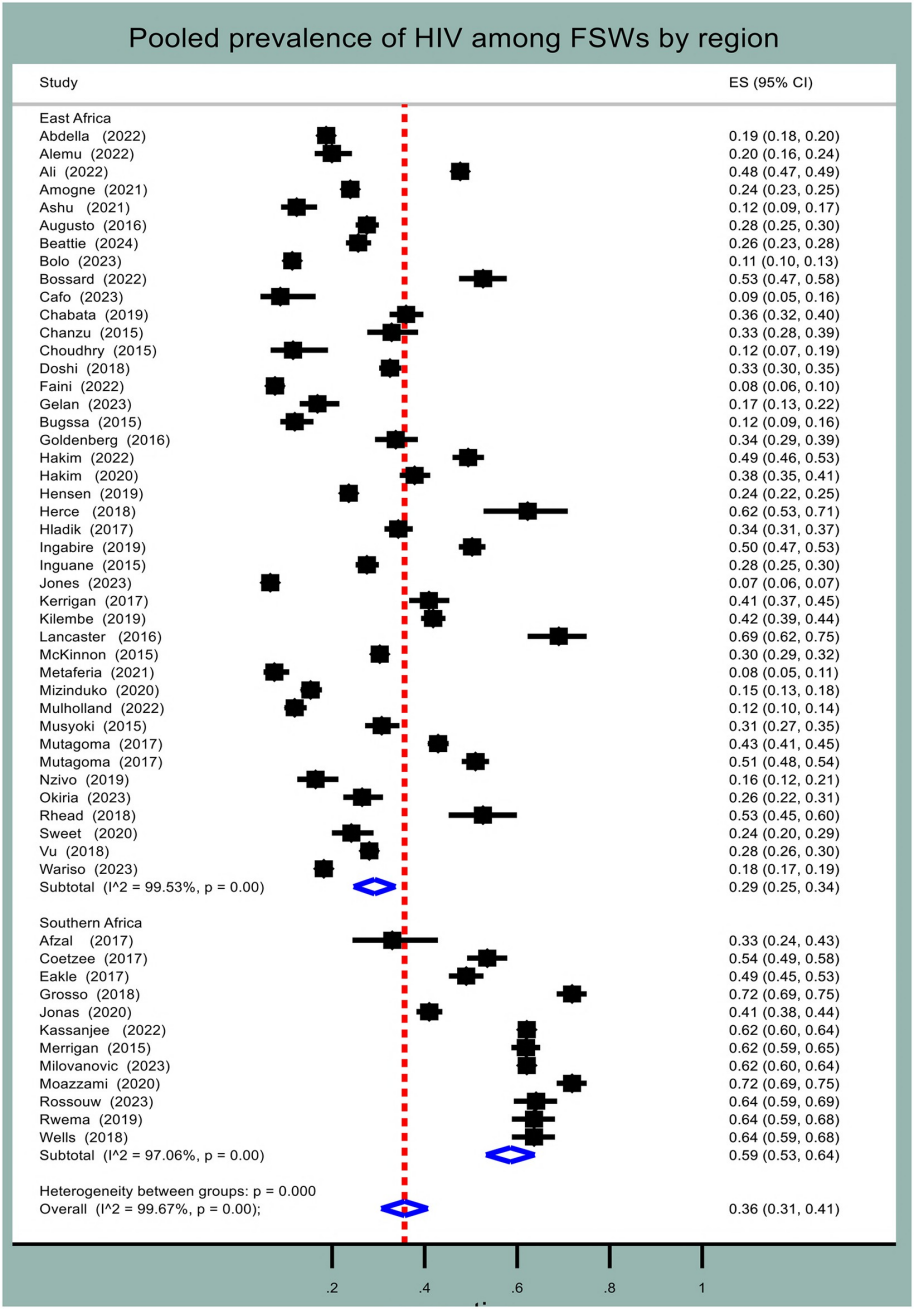
Forest plot for pooled prevalence of HIV among female sex workers in Eastern and Southern Africa by region.

In addition, subgroup analysis was done by region. The pooled prevalence of HIV among FSWs in Southern African countries was 59.0% (95% CI: 53.0%–64.0%, I^2^ = 97.1%, p-value < 0.001), while in Eastern African countries it was 29.0% (95% CI: 25.0%–34.0%, I^2^ = 99.5%, p-value < 0.001) (Fig 4).

Besides, HIV prevalence was assessed using study design. Based on this, the pooled prevalence of HIV was 37% (95% CI: 32.0%–42.0%, I^2^ = 99.5%, p-value < 0.001) among cross-sectional studies, whereas it was 23% (95% CI: 10.0%–37.0%, I^2^ = 99.8%, p-value < 0.001) in cohort studies (S1 File).

Furthermore, another subgroup analysis was also conducted using risk of bias. Thus, results showed that the pooled prevalence of HIV among female sex workers was 37.0% (95% CI: 25.0%–48.0%, I^2^ = 97.7%, p-value < 0.001) among studies with moderate risk of bias, whereas it was 36% (95% CI: 30.0%–41.0%, I^2^ = 99.7%, p-value < 0.001) (S1 File).

#### Sensitivity analysis

**Fig 5:**
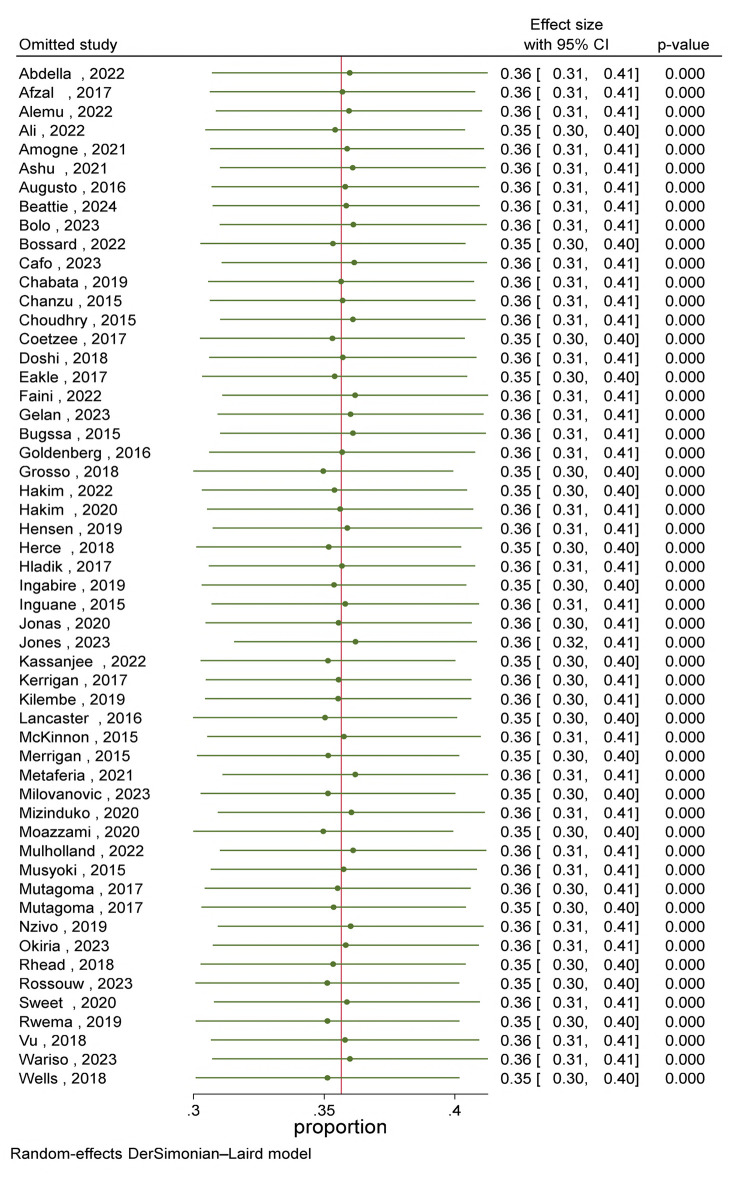
Sensitivity analysis forest plot for pooled prevalence of HIV among female sex workers in Eastern and Southern Africa.

Sensitivity analysis was conducted to detect influential studies on the pooled prevalence of HIV. Omitting studies using leave-one-out meta-analysis didn’t influence the overall prevalence of HIV (Fig 5).

### Factors associated with HIV among female sex workers

We conducted a meta-analysis for potential factors using the random effect DerSimonian-Laird model to identify the POR and its 95% confidence interval. Data for variables including age, marital status, educational status, STI, Syphilis, Neisseria Gonorrhea, Trichomonas Vaginalis, genital ulcer, Hepatitis B, condom breakage history, non-consistent condom use, alcohol, and injectable drug use before sex were extracted. A random effect model showed that age, marital status, educational status, STI (any type), syphilis, Hepatitis B infection, alcohol, and injectable drug use before sex were significantly associated with increased odds of HIV infection among FSWs in Eastern and Southern Africa.

The odds of HIV among FSWs whose age ≥ 35 and between 25–34 years were 7.35 times (POR = 7.35; 95% CI: 5.26, 10.28) and 2.91 times (POR = 2.91; 95% CI: 2.02, 4.21) higher than those FSWs whose age was <25 years, respectively.

Being married, divorced, and widowed was found to be significantly associated with increased odds of HIV infection as compared to being single (never married). The odds of HIV among married, divorced, and widowed FSWs were 1.33 times (POR = 1.33; 95% CI: 1.07, 1.66), 1.72 times (POR = 1.72; 95% CI: 1.39, 2.13), and 2.70 times (POR = 2.70; 95% CI: 2.09, 3.49) higher than the odds of HIV among single FSWs, respectively.

Female sex workers who had primary education had 29% higher odds of HIV (POR = 1.29; 95% CI: 1.08, 1.55) as compared to those FSWs who had higher education.

The association between STI and HIV was also examined by pooling the results of nine studies. Female sex workers who had STI (any type) had 51% increased odds of HIV (POR = 1.51; 95% CI: 1.20, 1.90) as compared to those who hadn’t STI.

A significant association between syphilis and HIV was also identified. FSWs who were reactive for syphilis had 2.57 times higher odds of HIV (POR = 2.57; 95% CI: 1.66, 3.98) as compared to those who were non-reactive.

Those FSWs positive for Hepatitis B had 2.6 times higher odds of HIV (POR = 2.60; 95% CI: 1.07, 6.32) than those of negative individuals. In the narrative synthesis, participants who were positive for Hepatitis C infection had 10.83 times higher odds of HIV (OR = 10.83; 95% CI: 4.65, 25.20) than their counterparts [36].

The use of alcohol and injectable drugs before sex was significantly associated with HIV among FSWs. Those who drink alcohol and use injectable drugs had 21% (POR = 1.21; 95% CI: 1.01, 1.45) and 75% (POR = 1.75; 95% CI: 1.24, 2.47) increased odds of HIV as compared to their counterparts, respectively (Table 2).

**Table 2:** Factors associated with HIV infection among FSWs in Eastern and Southern Africa.

Variables	Exposure	Comparator	Number of studies	POR (95% CI)	I^2^, p-value
**Age in years**	≥35	<25	6	7.35(5.26, 10.28)	39.2%, 0.144
	25–34	<25	6	2.91(2.02, 4.21)	74.0%, 0.002
**Marital status**	Married	Single	10	1.33(1.07, 1.66)	46.2%, 0.053
	Divorced	Single	9	1.72(1.39, 2.13)	74.3%, <0.001
	Widowed	Single	4	2.70(2.09, 3.49)	31.2%, 0.225
**Educational status**	No education	Higher	4	1.67(0.90, 3.08)	87.8%, <0.001
	Primary	Higher	2	1.29(1.08, 1.55)	0.0%, 0.769
**STI (any type)**	Yes	No	9	1.51(1.20, 1.90)	86.8%, <0.001
**Syphilis**	Yes	No	8	2.57(1.66, 3.98)	85.8%, <0.001
**Neisseria Gonorrhea**	Yes	No	2	2.79(0.81,9.58)	80.9%, 0.022
**Trichomonas vaginalis**	Yes	No	2	1.30(0.74, 2.30)	84.9%, 0.010
**Genital ulcer**	Yes	No	2	2.59 (0.72, 9.29)	71.9%, 0.059
**Hepatitis B**	Yes	No	2	2.60(1.07,6.32)	0.0%, 0.627
**Condom breakage**	Yes	No	4	1.45(0.94, 2.24)	77.9%, 0.011
**Non-consistent condom use**	Yes	No	2	1.90(0.54,6.66)	88.9%, <0.001
**Alcohol use before sex**	Yes	No	5	1.21(1.01, 1.45)	42.4%, 0.139
**Injectable drug use**	Yes	No	4	1.75(1.24, 2.47)	37.8%, 0.185

## Discussion

Female sex workers have been identified as key affected population groups for HIV infection in Eastern and Southern Africa. However, to our knowledge, the pooled prevalence of HIV infection among these groups had not been documented. Therefore, by this study, the pooled prevalence of HIV and its associated factors among FSWs in Eastern and Southern Africa was determined.

The pooled prevalence of HIV among FSWs in Eastern and Southern Africa (36.0%, 95% CI: 31.0–41.0%) is disproportionately higher than findings from high-income regions such as the United States (17.3%, 95% CI: 13.5–21.9%) [20] and Europe (<1%) [86], middle-income countries like Brazil (5.3%, 95% CI: 4.4–6.2%) [87], and Thailand (20.2%, 95% CI: 16.3–24.7%) [88]. In addition, a systematic review and meta-analysis reports in low- and middle-income countries (11.8%, 95% CI: 11.6–12.0%) [14], and the Middle East and North Africa (1.4%, 95% CI: 1.1–1.8%) [89] showed a lower prevalence of HIV in FSWs as compared to this study finding. The higher prevalence in Eastern and Southern Africa can be explained by the pronounced impact of poverty, stigma, and discrimination towards sex work and HIV in developing countries by creating barriers to accessing HIV prevention, detection, and treatment services [13,90]. The higher incidence and prevalence of HIV in the general population of the study setting can also be a reason for this higher prevalence among FSWs since their clients are from the population [1]. The observed differences in HIV prevalence among FSWs across regions and countries could be due to the variations in socio-economic status, culture, and level of implementation of the prevention strategy [27]. The disproportionately high prevalence of HIV among FSWs in Eastern and Southern Africa requires concerted efforts in the region to target the potential sources of HIV infection [18].

In this systematic review, significant heterogeneity is observed across the included studies. However, there is no single study that adequately describes the prevalence of HIV among FSWs in Eastern and Southern Africa. Attempts were made to determine whether the observed differences across the included studies might be explained by differences in time, geography, study design, and risk of bias. During our sensitivity analysis, an influential study on the overall estimate wasn’t detected. Previous systematic review and meta-analysis reports in other regions of the world have also shown high heterogeneity [20].

The pooled prevalence of HIV among FSWs in the region significantly decreased within the last 10 years. This might be due to improvements in HIV diagnosis, treatment, and virological suppression by many countries in Eastern and Southern Africa [91,92]. Progresses in HIV prevention, care, and treatment programs during the triple 90 and 95 targets introduced by UNAIDS might be possible reasons for the decline in HIV prevalence over time [93]. The higher prevalence of HIV in Southern African countries might be attributed to the existing socio-economic, behavioral, and structural factors that favor HIV infection to a higher extent than the Eastern African countries, including poverty, inconsistent use of condoms, harmful norms regarding gender and prostitution, sexual violence, alcohol, and substance use as identified in studies done in Southern Africa [94–98].

The positive association between older age and HIV infection is supported by evidence from Togo [11], Brazil [87], and a systematic review from India [99]. This can be attributed to the increased probability of exposure to risky sexual behaviors among older age groups due to their longer stay in sex work that prone the FSWs to repeated violence, inconsistent condom use, addiction to alcohol and injectable drugs, and negligence for preventive measures. Likewise, FSWs who are or have been married, divorced, or widowed face a higher risk of HIV infection as compared to single FSWs. This finding is supported by studies from Ghana [100] and Ethiopia [101] that found HIV preventive practices significantly common in single FSWs. This can be due to differences in healthcare decision-making autonomy. Single FSWs are confident enough to utilize HIV protective services and methods compared to married FSWs due to their freedom in decision-making. Women who have history of marriage may have been exposed to unprotected sex during their marital relationship, making them less familiar to consistent use of condoms and other protective methods when engaging in sex work.

In this study, those FSWs who had primary education had an increased odds of HIV as compared to those who had higher education. This finding was consistent with a study conducted in Brazil [87] and a systematic review in India [99]. A study from Bangladesh indicates that school-based HIV/AIDS education is one of the proven intervention strategies for providing information regarding HIV/AIDS transmission methods, prevention mechanisms, and its consequences [102]. A study from Ethiopia regarding HIV preventive practice indicates that longer stays in school lead to behavioral changes enhancing HIV preventive practices [103].

History of STIs significantly increases the odds of HIV infection in FSWs. This finding is supported by previous evidence [104]. Specifically, those FSWs who were reactive for syphilis had higher odds of HIV as compared to non-reactive women. This finding is supported by a systematic review and meta-analysis that estimate the effect of syphilis on HIV acquisition [105] and a study conducted in Ethiopia [106]. Mucosal ulceration, disruption, recruitment of HIV target cells to the genital mucosa following ulceration, and inflammation caused by STI pathogens have been identified as a reason for increased risk of HIV among those who had STI [107–109]. Hepatitis B virus infection is significantly associated with HIV infection. This can be because of the common transmission mechanisms of the two infections, including injectable drug use, unprotected sexual intercourse, and multiple sexual partners [110,111]. This implies the need for integrated preventive services in FSWs that target both viruses simultaneously.

In our finding, the significant association observed between alcohol, injectable drug use before sex and HIV infection might be due to the high involvement in risky sexual practices and violence experiences among FSWs with these behaviours. According to a previous study, drinking alcohol increases the likelihood of engaging in anal and condom-free sex [112]. Injectable drug use increases the risk of HIV infection due to sharing needles. This finding is supported by studies from India [99] and Indonesia [113]. Women who use injectable drugs have a higher risk of experiencing sexual abuse or rape by their sexual partners in addition to sharing contaminated syringes [114,115].

### Implications of the study

The implications of the study were seen based on hallmarks of public health, including social justice, prevention, and population focus. FSWs in Eastern and Southern African countries are highly affected key groups by HIV in terms of population focus of public health. Regarding social justice, HIV treatment, care, and preventive services should be distributed equitably to reach these marginalised populations. From the prevention perspective of public health, FSWs should be targeted for intensive preventive services focusing on behavioral change and minimising structural barriers.

### Strengths and limitations of the study

Strengths: This study was done in the world’s highest HIV burden region (Eastern and Southern Africa) to estimate the pooled prevalence and associated factors of HIV among FSWs, which could help concerned bodies like the World Health Organization (WHO), UNAIDS, governments and non-governmental organizations in the region to provide area-targeted interventions through setting priorities. In addition, studies that estimate the prevalence of HIV based on blood test results using biological assays were included, while studies that estimate HIV prevalence based on self-report of FSWs were excluded. This helps to eliminate recall and social desirability bias in the pooled HIV estimates. Besides, the majority of the included studies have a low risk of bias and a large sample size that might improve the generalizability of study findings.

Limitations: Even though subgroup and sensitivity analysis were conducted accordingly, the level of heterogeneity across studies was considerable. In addition, primary studies were not conducted in all countries located in Eastern and Southern African countries. These issues might limit the generalizability of our study findings. Hence, interpretations of findings should consider the limitations of the study.

## Conclusion and recommendations

The prevalence of HIV among FSWs in Eastern and Southern African countries is unacceptably high. Nearly three-tenths (29%) and six-tenths (59%) of FSWs in Eastern and Southern African countries have HIV infection, respectively. Within a decade (2015–2024), 11% decrement of HIV prevalence among FSWs has been achieved in the region.

Sociodemographic factors, including being older, married, divorced, widowed, and having a low level of education, were found significantly associated with an increased risk of HIV infection. In addition, STI, alcohol, and drug use before sex were significantly associated with an increased risk of HIV infection. The findings of this study suggest an urgent need to improve access to quality HIV prevention and treatment services for FSWs in Eastern and Southern Africa. Therefore, WHO, UNAIDS, and their stakeholders need to give intervention priority to FSWs in the region by providing behavioral interventions to reduce alcohol and injectable drug use. We recommend governments and non-governmental organizations in the region to enhance access to healthcare services tailored for FSWs, while also strengthening psychological, social, and legal support networks to reduce stigma and ensure their protection. Creating partnerships with international organizations will help governments to pool resources for effective and sustainable interventions. Clinicians could facilitate early detection and treatment of STIs and Hepatitis B virus infection.

## Supporting information

S1 File Supporting information for a systematic review and meta-analysis on the prevalence and associated factors of HIV among female sex workers in Eastern and Southern Africa. (PDF)

S2 File Data used for meta-analysis on the prevalence and associated factors of HIV among female sex workers in Eastern and Southern Africa. (XLSX)

S3 File Preferred Reporting Items for Systematic Reviews and Meta-Analyses (PRISMA) 2020 statement. (DOCX)

## Abbreviations

AIDS: Acquired Immunodeficiency Syndrome
FSWs: Female Sex Workers
HIV: Human Immunodeficiency Virus
PRISMA: Preferred Reporting Items for Systematic Review and Meta-Analysis
POR: Pooled Odds Ratio
STI: Sexually Transmitted Infection
UNAIDS: Joint United Nations Programme on HIV/AIDS
WHO: World Health Organization
