# Supplementary material for: Prevalence and associated factors of HIV among female sex workers in Eastern and Southern Africa: Systematic review and meta-analysis
Source: PLoS One. 2024 Dec 2;19(12):e0313868. doi: 10.1371/journal.pone.0313868 (PMC11611193; doi:10.1371/journal.pone.0313868)
Supplement: S1 File — (PDF) [file pone.0313868.s001.pdf]

Supplementary Table 1. Searching strategy utilized in different databases to conduct systematic review and meta-analysis on pooled prevalence and associated factors of HIV among female sex workers in Eastern and Southern Africa.

**Database and number of articles retrieved**	**Combination**
PubMed=1980	(((((Prevalence OR Magnitude OR Proportion OR burden OR Epidemiology) AND (HIV OR HIV/AIDS OR AIDS Virus OR Acquired Immune Deficiency Syndrome Virus)) OR (HIV OR HIV/AIDS OR AIDS Virus OR Acquired Immune Deficiency Syndrome Virus[MeSH Terms])) AND (Female Sex worker OR commercial sex worker OR sex worker OR prostitute OR brothel worker OR transaction* sex worker)) AND (Factors OR “Associated factors” OR Determinants OR Predictors OR “Related factors” OR “Risk factor*”)) AND (Eritrea OR Ethiopia OR Djibouti OR Somalia OR South Sudan OR Kenya OR Uganda OR Rwanda OR Burundi OR Tanzania OR Malawi OR Zambia OR Zimbabwe OR Mozambique OR Seychelles OR Comoros OR Madagascar OR Mauritius OR Eswatini OR Swaziland OR Lesotho OR Botswana OR South Africa OR Namibia)<br>Year: January 1, 2015 to March 30, 2024<br>Language: English
Embase =312	('prevalence' OR 'magnitude' OR 'proportion' OR 'burden' OR 'epidemiology') AND ('hiv' OR 'hiv/aids' OR 'aids virus' OR 'acquired immune deficiency syndrome virus' OR 'acquired immunodeficiency syndrome virus' OR 'htlv-iii' OR 'human immunodeficiency virus' OR 'human immunodeficiency viruses' OR 'human t cell lymphotropic virus type iii' OR 'human t lymphotropic virus type iii' OR 'human t-cell leukemia virus type iii' OR 'human t-cell lymphotropic virus type iii' OR 'human t-lymphotropic virus type iii' OR 'immunodeficiency virus, human' OR 'immunodeficiency viruses, human' OR 'lav-htlv-iii' OR 'lymphadenopathy-associated virus' OR 'virus, human immunodeficiency' OR 'viruses, human immunodeficiency') AND ('female sex worker' OR 'commercial sex worker' OR 'sex worker' OR 'prostitute' OR 'brothel worker' OR 'transaction* sex worker') AND ('factors' OR 'associated factors' OR 'determinants' OR 'predictors' OR 'related factors' OR 'risk factor') AND ('eritrea' OR 'ethiopia' OR 'djibouti' OR 'somalia' OR 'south sudan' OR 'kenya' OR 'uganda' OR 'rwanda' OR 'burundi' OR 'tanzania' OR 'malawi' OR 'zambia' OR 'zimbabwe' OR 'mozambique' OR 'seychelles' OR 'comoros' OR 'madagascar' OR 'mauritius' OR 'eswatini' OR 'swaziland' OR 'lesotho' OR 'botswana' OR 'south africa' OR 'namibia') AND [english]/lim AND [01-01-2015]/sd NOT [31-03-2024]/sd AND [2015-2024]/py
**Scopus=2580**	**(prevalence OR magnitude OR proportion OR burden OR epidemiology) AND (hiv OR hiv/aids OR aids AND virus OR acquired AND immune AND deficiency AND syndrome AND virus) AND (female AND sex AND worker OR commercial AND sex AND worker OR sex AND worker OR prostitute OR brothel AND worker OR transaction* AND sex AND worker) AND (factors OR "Associated factors" OR determinants OR predictors OR "Related factors" OR "Risk factor*") AND (eritrea OR ethiopia OR djibouti OR somalia OR south AND sudan**

	**OR kenya OR uganda OR rwanda OR burundi OR tanzania OR malawi OR zambia OR zimbabwe OR mozambique OR seychelles OR comoros OR madagascar OR mauritius OR eswatini OR swaziland OR lesotho OR botswana OR south AND africa OR namibia) AND (LIMIT-TO (PUBYEAR , 2015) OR LIMIT-TO (PUBYEAR , 2016) OR LIMIT-TO (PUBYEAR , 2017) OR LIMIT-TO (PUBYEAR , 2018) OR LIMIT-TO (PUBYEAR , 2019) OR LIMIT-TO (PUBYEAR , 2020) OR LIMIT-TO (PUBYEAR , 2021) OR LIMIT-TO (PUBYEAR , 2022) OR LIMIT-TO (PUBYEAR , 2023) OR LIMIT-TO (PUBYEAR , 2024)) AND (LIMIT-TO (DOCTYPE , "ar")) AND (LIMIT-TO (LANGUAGE , "English"))**
ScienceDirect=1001	(prevalence OR magnitude OR proportion) AND (HIV infection OR HIV/AIDS OR “human immunodeficiency virus”) AND (“female sex worker” OR “Commercial sex worker” OR “sex worker”)<br>Additional filters; year: 2015-2024, type: research article; language: English
Hinari through research4life=696	(\(HIV among female sex workers\) AND \(Prevalence OR Magnitude OR Proportion\) AND \(Eritrea OR Ethiopia OR Djibouti OR Somalia OR South Sudan OR Kenya OR Uganda OR Rwanda OR Burundi OR Tanzania OR Malawi OR Zambia OR Zimbabwe OR Mozambique OR Seychelles OR Comoros OR Madagascar OR Mauritius OR Eswatini OR Swaziland OR Lesotho OR Botswana OR South Africa OR Namibia\))<br>Additional filters; date January 01, 2015-march 30/2024; language: English; type: journal article
AJOL =199	((Prevalence OR Magnitude OR Proportion) AND (HIV OR HIV/AIDS OR AIDS virus OR Human immunodeficiency virus OR Acquired immunodeficiency syndrome virus) AND (Female Sex worker OR commercial sex worker OR sex worker OR prostitute OR brothel worker OR transactional sex) AND (factors OR associated factors OR determinants OR Predictors OR Related factors OR Risk factors) AND (Eritrea OR Ethiopia OR Djibouti OR Somalia OR South Sudan OR Kenya OR Uganda OR Rwanda OR Burundi OR Tanzania OR Malawi OR Zambia OR Zimbabwe OR Mozambique OR Seychelles OR Comoros OR Madagascar OR Mauritius OR Eswatini OR Swaziland OR Lesotho OR Botswana OR South Africa OR Namibia))

Supplementary Table 2. Study quality (risk of bias) assessment using Joanna Brigg’s Institute checklist for cross-sectional and cohort studies to conduct systematic review and meta-analysis on pooled prevalence and associated factors of HIV among female sex workers in Eastern and Southern Africa.

**Authors**	**Study design**	**Checklist Items (1-8 for cross-sectional studies, 1-11 for cohort studies)**											**Risk of Bias**
		1	2	3	4	5	6	7	8	9	10	11	
Abdella *et al*	cross-sectional	yes	yes	yes	yes	yes	yes	yes	yes				Low
Afzal *et al*	cross-sectional	no	yes	yes	yes	no	yes	unclear	yes				Moderate
Alemu *et al*	cross-sectional	yes	yes	yes	yes	yes	yes	yes	yes				Low

Ali et al	cross-sectional	yes	yes	yes	yes	yes	yes	unclear	yes				Low
Amogne *et al*	cross-sectional	yes	yes	yes	yes	yes	yes	yes	yes				Low
Ashu *et al*	cross-sectional	yes	yes	yes	yes	yes	yes	yes	yes				Low
Augusto *et al*	cross-sectional	yes	yes	yes	yes	yes	yes	yes	yes				Low
Beattie *et al*	cross-sectional	yes	yes	yes	yes	yes	yes	yes	yes				Low
Bolo *et al*	cross-sectional	yes	yes	yes	yes	yes	yes	yes	yes				Low
Bossard *et al*	cross-sectional	yes	yes	yes	yes	yes	yes	yes	yes				Low
Cafo *et al*	cross-sectional	Yes	unclear	yes	yes	no	yes	yes	yes				Moderate
Chabata *et al*	cross-sectional	yes	yes	yes	yes	yes	yes	yes	yes				Low
Chanzu *et al*	cross-sectional	yes	yes	yes	yes	yes	yes	yes	yes				Low
Choudhry *et al*	cross-sectional	yes	yes	yes	yes	yes	yes	yes	yes				Low
Coetzee *et al*	cross-sectional	yes	yes	yes	yes	yes	yes	yes	yes				Low
Doshi *et al*	cross-sectional	yes	no	yes	yes	yes	yes	yes	yes				Low
Eakle *et al*	cohort	yes	unclear	yes	yes	no	yes	yes	yes	yes	unclear	yes	Moderate
Faini *et al*	cohort	yes	yes	yes	yes	yes	yes	yes	yes	yes	yes	yes	Low
Gelan *et al*	cross-sectional	yes	yes	yes	yes	yes	yes	Unclear	yes				Low
Bugssa *et al*	cross-sectional	yes	yes	yes	yes	yes	yes	yes	yes				Low
Goldenberg *et al*	cross-sectional	yes	yes	unclear	yes	yes	yes	yes	yes				Low
Grosso *et al*	cross-sectional	yes	yes	yes	yes	yes	yes	yes	yes				Low
Hakim *et al*	cross-sectional	yes	yes	yes	yes	yes	yes	yes	yes				Low
Hakim et al	cross-sectional	yes	yes	yes	yes	yes	yes	yes	yes				Low
Hensen *et al*	cross-sectional	yes	yes	yes	yes	yes	yes	yes	yes				Low
Herce *et al*	cross-sectional	yes	yes	yes	yes	yes	yes	yes	yes				Low
Hladik *et al*	cross-sectional	yes	yes	yes	yes	yes	yes	yes	yes				Low
Ingabire *et al*	cross-sectional	yes	unclear	yes	unclear	yes	yes	yes	yes				Moderate
Inguane *et al*	cross-sectional	yes	yes	yes	unclear	yes	yes	yes	yes				Low
Jonas *et al*	cross-sectional	yes	unclear	yes	unclear	yes	yes	yes	yes				Moderate
Jones *et al*	cohort	yes	yes	yes	yes	yes	yes	yes	yes	no	unclear	yes	Low
Kassanjee *et al*	cross-sectional	yes	yes	yes	yes	yes	yes	yes	yes				Low
Kerrigan *et al*	cross-sectional	yes	yes	yes	yes	yes	yes	yes	yes				Low

Kilembe *et al*	cross-sectional	unclear	yes	yes	yes	yes	yes	yes	yes				Low
Lancaster *et al*	cross-sectional	yes	yes	no	yes	yes	yes	yes	yes				Low
McKinnon *et al*	cohort	yes	yes	yes	yes	yes	yes	yes	yes	yes	yes	yes	Low
Merrigan *et al*	cross-sectional	yes	yes	unclear	no	yes	yes	yes	yes				Low
Metaferia *et al*	cross-sectional	yes	yes	yes	yes	yes	yes	yes	yes				Low
Milovanovic *et al*	cross-sectional	yes	yes	yes	unclear	yes	yes	yes	yes				Low
Mizinduko *et al*	cross-sectional	yes	yes	yes	yes	yes	yes	yes	yes				Low
Moazzami *et al*	cross-sectional	yes	yes	yes	yes	yes	yes	yes	yes				Low
Mulholland *et al*	cross-sectional	yes	yes	yes	yes	yes	yes	unclear	yes				Low
Musyoki *et al*	cross-sectional	yes	yes	yes	yes	yes	yes	yes	yes				Low
Mutagoma *et al*	cross-sectional	yes	yes	yes	yes	yes	yes	yes	yes				Low
Mutagoma *et al*	cross-sectional	yes	yes	yes	unclear	yes	yes	yes	yes				Low
Nzivo *et al*	cross-sectional	yes	yes	yes	yes	yes	unclear	yes	yes				Low
Okiria *et al*	cross-sectional	yes	yes	yes	yes	yes	yes	yes	yes				Low
Rhead *et al*	cross-sectional	yes	yes	yes	yes	yes	yes	yes	yes				Low
Rossouw *et al*	cross-sectional	yes	yes	yes	yes	yes	yes	yes	yes				Low
Sweet *et al*	cohort	yes	yes	yes	no	yes	yes	yes	yes	yes	unclear	yes	Low
Rwema *et al*	cross-sectional	yes	yes	yes	yes	unclear	yes	yes	yes				Low
Vu *et al*	cross-sectional	yes	yes	yes	yes	yes	yes	yes	yes				Low
Wariso *et al*	cross-sectional	yes	yes	yes	yes	yes	yes	yes	yes				Low
Wells *et al*	cross-sectional	yes	yes	yes	yes	yes	yes	yes	yes				Low

JBI checklist items for cross-sectional studies

1 Were the criteria for inclusion in the sample clearly defined?
2 Were the study subjects and the setting described in detail?
3 Was the exposure measured in a valid and reliable way?
4 Were objective, standard criteria used for measurement of the condition?
5 Were confounding factors identified?
6 Were strategies to deal with confounding factors stated?
7 Were the outcomes measured in a valid and reliable way?
8 Was appropriate statistical analysis used?

JBI checklist items for cohort studies

1	Were the two groups similar and recruited from the same population?
2	Were the exposures measured similarly to assign people to both exposed and unexposed groups?
3	Was the exposure measured in a valid and reliable way?
4	Were confounding factors identified?
5	Were strategies to deal with confounding factors stated?
6	Were the groups/participants free of the outcome at the start of the study (or at the moment of exposure)?
7	Were the outcomes measured in a valid and reliable way?
8	Was the follow up time reported and sufficient to be long enough for outcomes to occur?
9	Was follow up complete, and if not, were the reasons to loss to follow up described and explored?
10	Were strategies to address incomplete follow up utilized?
11	Was appropriate statistical analysis used?

Interpretation of risk of bias

For cross-sectional studies (total items: 8): 7-8, low risk of bias; 4-6, moderate risk of bias; and 0-3, high risk of bias.

For cohort studies (total items: 11): 9-11, low risk of bias; 5-8, moderate low risk of bias; 0-4, high low risk of bias.

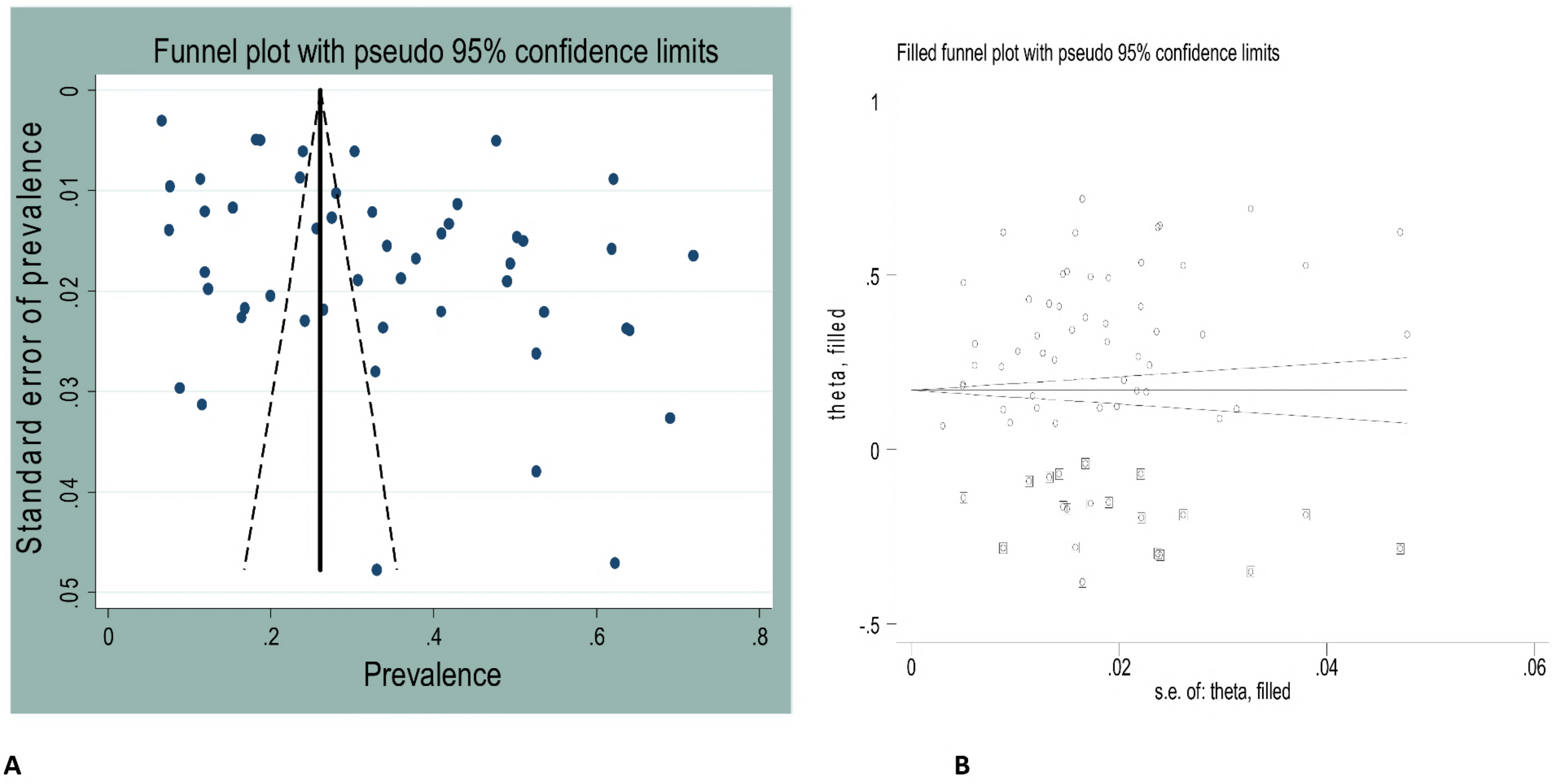

Supplementary Figure 1. Funnel plot evaluating the existence of publication bias (A), and trim-and-fill analysis to handle publication bias (B) for a systematic review and meta-analysis on pooled prevalence and associated factors of HIV among female sex workers in Eastern and Southern Africa.

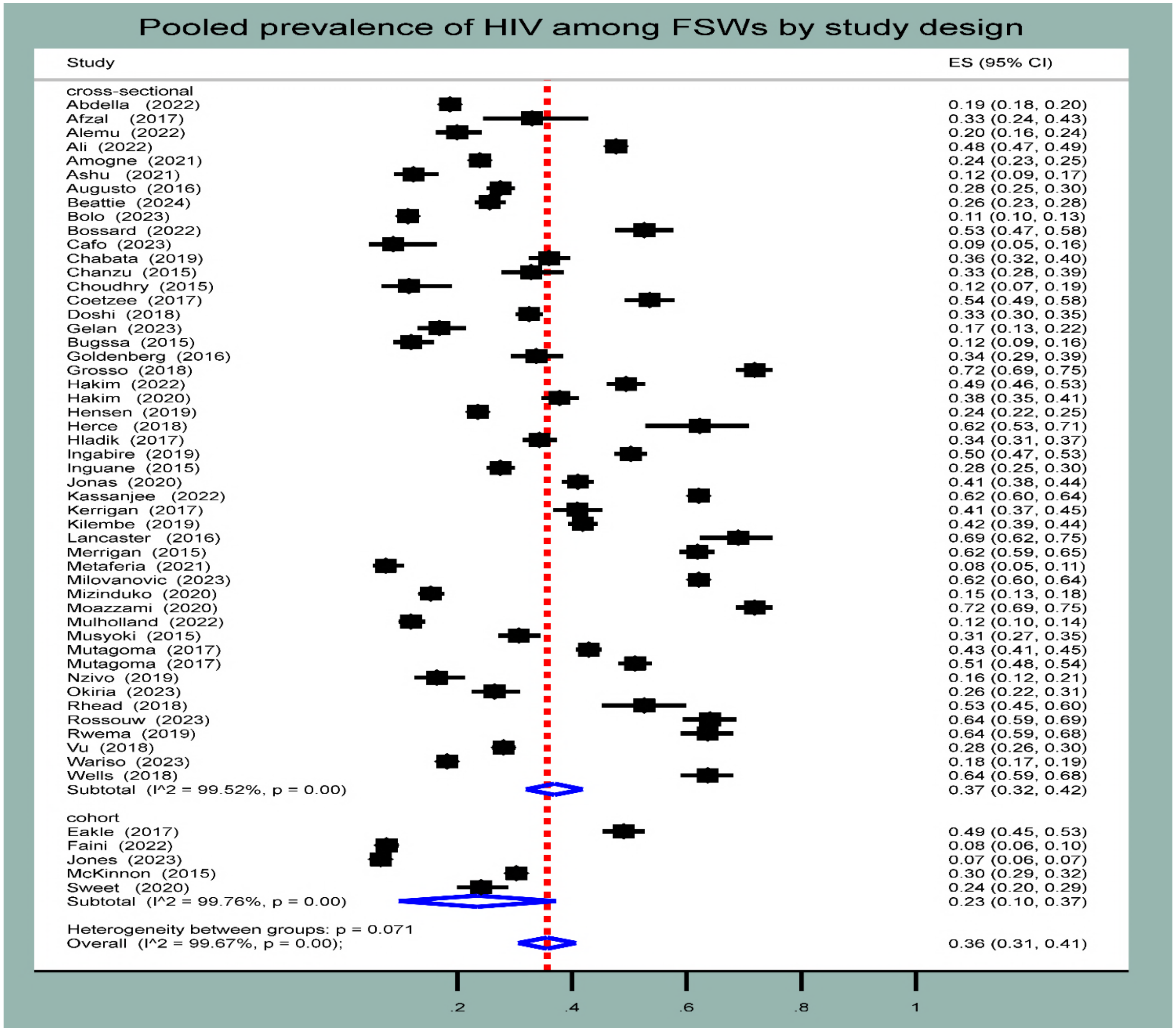

Supplementary Figure 2. Forest plot for pooled prevalence of HIV among female sex workers in Eastern and Southern Africa by study design.

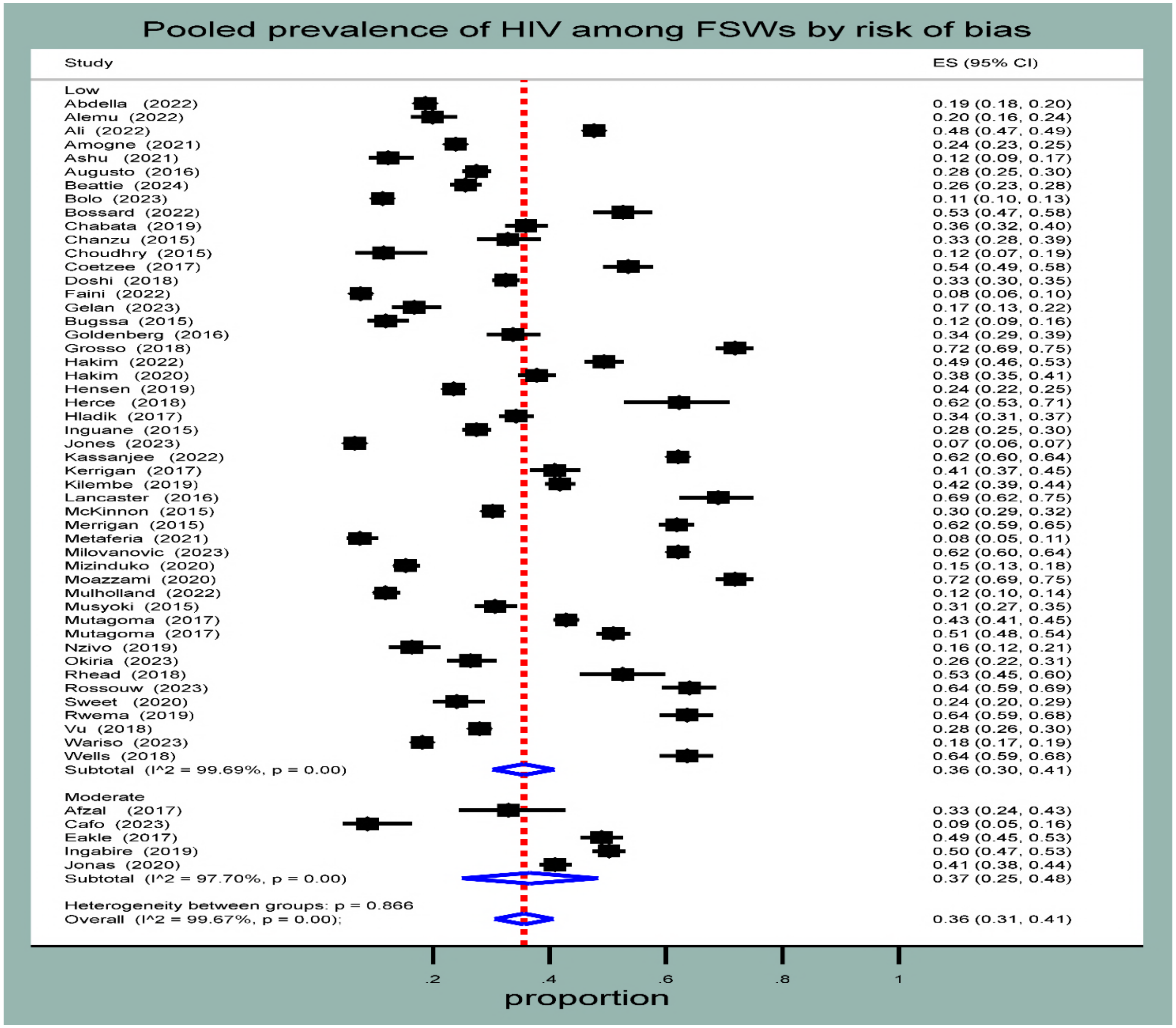

Supplementary Figure 3. Forest plot for pooled prevalence of HIV among female sex workers in Eastern and Southern Africa by study design.
